# Toll-Like Receptor mRNA Expression Is Selectively Increased in the Colonic Mucosa of Two Animal Models Relevant to Irritable Bowel Syndrome

**DOI:** 10.1371/journal.pone.0008226

**Published:** 2009-12-09

**Authors:** Declan P. McKernan, Aoife Nolan, Elizabeth K. Brint, Siobhain M. O'Mahony, Niall P. Hyland, John F. Cryan, Timothy G. Dinan

**Affiliations:** 1 Laboratory of Neurogastroenterology, Alimentary Pharmabiotic Centre, University College Cork, Cork, Ireland; 2 School of Pharmacy, University College Cork, Cork, Ireland; 3 Department of Psychiatry, University College Cork, Cork, Ireland; 4 Department of Pharmacology and Therapeutics, University College Cork, Cork, Ireland; 5 Department of Anatomy, University College Cork, Cork, Ireland; 6 Department of Pathology, University College Cork, Cork, Ireland; HelmholtzZentrum München, Germany

## Abstract

**Background:**

Irritable bowel syndrome (IBS) is largely viewed as a stress-related disorder caused by aberrant brain-gut–immune communication and altered gastrointestinal (GI) homeostasis. Accumulating evidence demonstrates that stress modulates innate immune responses; however, very little is known on the immunological effects of stress on the GI tract. Toll-like receptors (TLRs) are critical pattern recognition molecules of the innate immune system. Activation of TLRs by bacterial and viral molecules leads to activation of NF-kB and an increase in inflammatory cytokine expression. It was our hypothesis that innate immune receptor expression may be changed in the gastrointestinal tract of animals with stress-induced IBS-like symptoms.

**Methodology/Principal Findings:**

In this study, our objective was to evaluate the TLR expression profile in the colonic mucosa of two rat strains that display colonic visceral hypersensivity; the stress-sensitive Wistar-Kyoto (WKY) rat and the maternally separated (MS) rat. Quantitative PCR of TLR2-10 mRNA in both the proximal and distal colonic mucosae was carried out in adulthood. Significant increases are seen in the mRNA levels of TLR3, 4 & 5 in both the distal and proximal colonic mucosa of MS rats compared with controls. No significant differences were noted for TLR 2, 7, 9 & 10 while TLR 6 could not be detected in any samples in both rat strains. The WKY strain have increased levels of mRNA expression of TLR3, 4, 5, 7, 8, 9 & 10 in both the distal and proximal colonic mucosa compared to the control Sprague-Dawley strain. No significant differences in expression were found for TLR2 while as before TLR6 could not be detected in all samples in both strains.

**Conclusions:**

These data suggest that both early life stress (MS) and a genetic predisposition (WKY) to stress affect the expression of key sentinels of the innate immune system which may have direct relevance for the molecular pathophysiology of IBS.

## Introduction

Irritable Bowel Syndrome (IBS) is a highly prevalent functional disorder of the gastrointestinal tract characterized by the presence of abdominal pain or discomfort, an alteration in bowel habit and the absence of reproducible biomarkers [1], [2]. It can result in significant impairment in quality of life and in psychological function. The precise molecular pathophysiology remains to be elucidated but altered visceral perception (visceral hypersensitivity) and gut dysmotility are important contributors to symptom progression [3], [4]. IBS is generally viewed as a disorder of the brain-gut axis with stress playing an important role [5], [6]. Moreover, accumulating evidence demonstrates that IBS patients display an over-activation of the hypothalamic-pituitary adrenal (HPA) axis which is crucial for integration of the body's response to stress [7].

Recently, we and others, have reported immune dysfunction in IBS patients [7]–[10]. Systemic inflammation is known to activate the HPA axis [11] and may account for the exaggerated HPA axis response reported in IBS such as the elevations in the inflammatory cytokine interleukin-6 (IL-6) [7]. Furthermore, it has also been shown that baseline levels of tumour necrosis factor α (TNFα), interleukin-1β (IL-1β) and IL-6 were elevated in diarrhoea predominant IBS patients (D-IBS) [10]. It is not known what causes this elevation in pro-inflammatory cytokines in the absence of visible signs of infection or inflammation; this is in contrast to post-infectious IBS (PI-IBS), where IBS symptoms develop following a gastrointestinal infection. This is thought to be due to residual inflammation post-infection and persistent changes in the colonic mucosal immune system and microbiota [12].

Toll-like receptors (TLRs) are members of the pattern recognition receptor family and play a central role in the initiation of innate cellular responses and the subsequent adaptive immune response to a variety of pathogens. There are ten TLRs in humans and they recognise different microbial ligands during infection [13]. There is also growing evidence to indicate that certain TLRs also sense products of damaged tissue [14]. Activation of the TLRs by either pathogenic ligand or host factors results in activation of downstream transcription factors such as NF-κB and IRF-3 resulting in expression of immune and inflammatory genes. TLRs are activated by various components of both bacterial and viral cell components e.g. TLR4 binds lipopolysaccharide (LPS) in gram-negative bacteria and TLR7 binds single stranded RNA (ssRNA) from viruses. Following receptor binding, a cellular protein cascade is initiated which results in the activation of NF-κB amongst other transcription factors, which in turn transcribe inflammatory cytokines [15]–[17]. TLRs are present on a variety of cell types and are found on colonic mucosal surfaces [18]. Few studies have investigated the impact of chronic stress on TLR expression and function, however of those that have done so, repeated social defeat stress has been shown to increase the expression of TLR2 and TLR4 [19] and increase TLR-mediated release of pro-inflammatory cytokines from splenic monocytes and dendritic cells [20]. Chronic restraint stress has also been shown to increase the expression of TLR4 in the spleen [21] and promotes immune suppression via PI3K signalling [22]. In this study, our objective was to examine the TLR expression profile in the colonic mucosae of two chronic stress models, the maternal separation (MS) model and Wistar-Kyoto (WKY) rats. MS rats show increased visceral hypersensitivity, increases in pro-inflammatory cytokine secretion following LPS stimulation and differences in the intestinal microbiota when compared to non separated (NS) rats [23]. Furthermore, MS rats have increased colonic permeability and increased mast cell protease release and number in the colonic mucosa [24], [25]. This mast cell activity was subsequently shown to occur in close apposition to enteric nerves [25], which is similar to that seen in IBS patients [24]. The WKY rat is a selectively bred strain that is hyper-responsive to stress [26], [27]. In particular, WKY rats have marked elevations in stress- and depression-associated changes when compared to Sprague Dawley (SD) rats, the strain most commonly used as a control comparator [28]. In addition to the depressed and anxious phenotype, increased sensitivity to colorectal distension (CRD) has been reported [23], [29], [30] coupled with alterations in colonic and gastric accommodation [31], [32] and colonic morphology [33] and function [34], [35] further supporting the use of these animals as a model relevant to IBS. Given that both MS and WKY rats are purported to have alterations in their stress and immune responses in addition to IBS-related behavioural and visceral hypersensitivity changes, we hypothesised that there could be changes in innate immune receptor expression in the gastrointestinal tract. We therefore investigated whether there were any perturbations in TLR mRNA in the colonic mucosa in both models.

## Materials and Methods

### Animals

Male SD and WKY rats (200–250 g,) were obtained from Harlan UK. Animals were allowed to acclimatise to our animal facility for at least one week. Temperature and light (12 hr light: 12 hr dark) were controlled, while intake of food and water were provided *ad libitum*. MS of facility reared SD rats was carried out from P2-P12 for 3 h per day as previously described [23]. Briefly, the MS rats were removed from their home cages at P2 and placed into plastic cages maintained at 30–33°C, in a separate room. Separations lasted for 3 h/day until P12 from 9:00 am until 12:00 pm. The NS groups were left undisturbed with their mothers.

Experimental group sizes were determined by a power calculation to detect differences at the 0.05 level. Following euthanasia by inhalable anaesthetic (isoflurane 2%) followed by decapitation; colonic mucosal scrapings (n = 5 per group) from distal and proximal colonic mucosa were collected at ∼8 weeks of age. Given that the distal and proximal colon contribute to different physiological functions the mucosae from each was analysed separately to assess if there also differed in their expression of TLRs. Specifically, the proximal colon stores food/faeces and has a major function in reabsorbing water and electrolytes whereas the distal colon has a greater role in the propulsion of the faeces out of the colon, in addition to fluid absorption [36]. Mucosal scrapings were stored in RNA Later (Ambion, Warrington, UK) at −80°C for analysis at a later date. Approval for this study was granted by University College Cork's Animal Experimentation Ethics Committee (Ethics Approval number & date).

### Sample Preparation

Colonic mucosal scrapings were homogenized using a Polytron PT2100 in RNA lysis buffer until all solids were lysed (Stratagene, La Jolla, USA). RNA extraction was carried out using the Stratagene Absolutely RNA® Miniprep kit according to manufacturer's instructions. Briefly, nucleic acids were extracted using a buffer and spin column protocol. The nucleic acids were then washed and separated using an elution column. DNase treatment subsequently removed any DNA and was carried out using the Ambion Turbo DNase kit (Ambion, Warrington, UK) according to manufacturer's instructions. RNA was quantified using NanoDrop™ spectrophotometer according to the manufacturer's instructions. RNA quality was assessed using the Agilent™ Bioanalyzer (Agilent, Stockport, UK) according to the manufacturer's procedure and an RNA integrity number (RIN) was calculated. RNA with RIN value >7 was used for subsequent experiments. RNA was reverse transcribed to cDNA using the Applied Biosystems™ High Capacity cDNA kit (Applied Biosystems, Warrington, UK) according to manufacturer's instructions. Briefly, Multiscribe Reverse Transcriptase (50 U/µL) was added as part of RT master mix, incubated for 25°C for 10 mins, 37°C for 2 hrs, 85°C for 5 mins and stored at 4°C.

### Quantitative Real-Time PCR

Quantitative PCR (Q-PCR) was carried out using probes (6 carboxy fluorescein - FAM) designed by Applied Biosystems™ to rat specific Toll-like receptors (TLR) 2–10 while using β-Actin as an endogenous control. Q-PCR was carried out on the ABI7300 Real Time PCR machine (Applied Biosystems, Warrington, UK). Samples were heated to 95°C for 10 mins, and then subjected to 40 cycles of amplification by melting at 95°C and annealing at 60°C for 1 min. Experimental samples were run in triplicate with 2 µL cDNA per reaction. To check for amplicon contamination, each run contained no template controls in triplicate for each probe used. Cycle threshold (Ct) values were recorded. Data was normalised using β-Actin and transformed using the 2^−ΔCt^ method [37].

### Statistical Analysis

Data are expressed as mean +/− SEM. Statistical differences were determined using Student's t-test with a correction for multiple comparisons. All tests were performed using GraphPad Prism 4 statistical software. Statistical significance was indicated as follows: * indicates p<0.05; ** indicates p<0.01 & *** indicates p<0.001.

## Results

### TLR Expression in Maternally Separated (MS) Compared with Non-Separated (NS) Tissues

**Figure 1:**
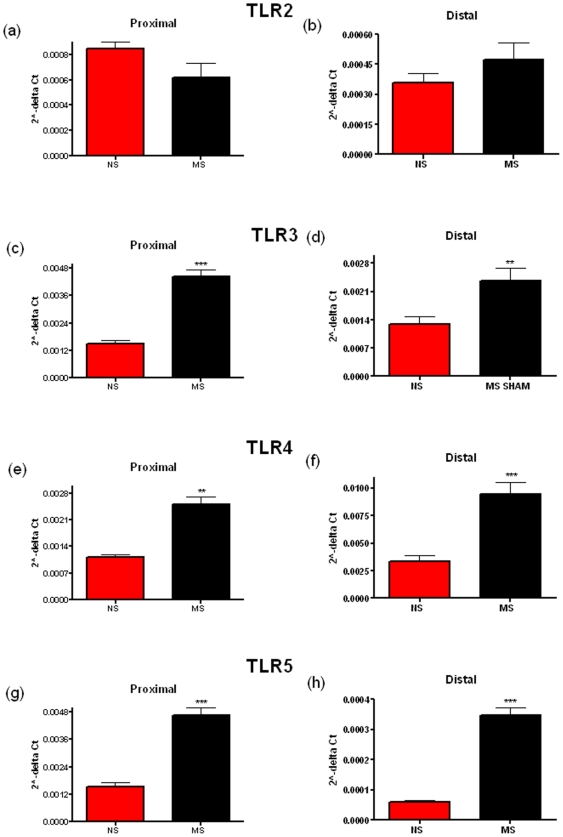
Relative levels of Toll-like Receptor (TLR) mRNA in the distal and proximal colonic mucosa of non-separated (NS) and maternally-separated (MS) Sprague-Dawley rats. TLR2 (a, b), TLR3 (c, d), TLR4 (e, f) & TLR5 (g, h) mRNA levels are shown in both the proximal (a, c, e & g) and the distal (b, d, f & h) colon. Data was normalized to β-Actin and expressed using the 2^−ΔCt^ method. (** indicates p<0.01, *** indicates p<0.001).

**Figure 2:**
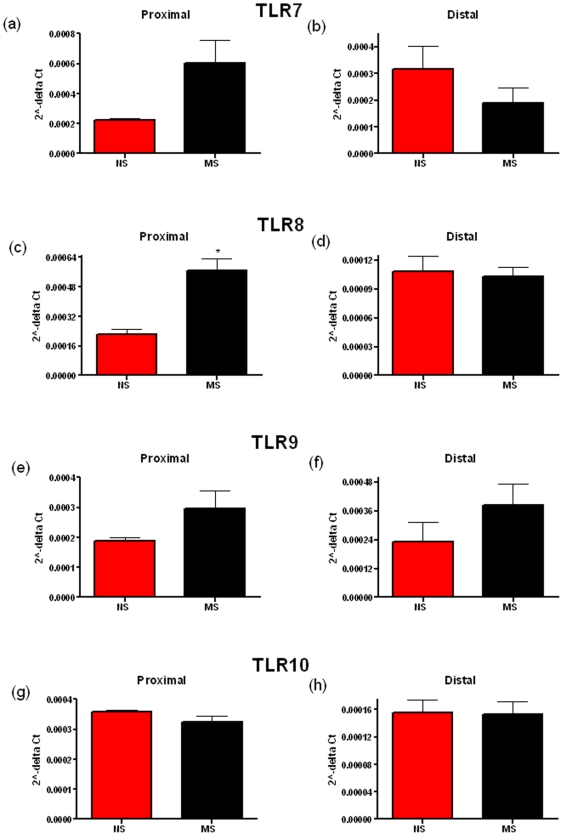
Relative levels of Toll-like Receptor (TLR) mRNA in the distal and proximal colonic mucosa of non-separated (NS) and maternally-separated (MS) Sprague-Dawley rats. TLR7 (a, b), TLR8 (c, d), TLR9 (e, f) & TLR10 (g, h) mRNA levels are shown in both the proximal (a, c, e & g) and the distal (b, d, f & h) colon. Data was normalized to β-Actin and expressed using the 2^−ΔCt^ method. (** indicates p<0.01, *** indicates p<0.001).

Early life stress causes perturbations in brain gut-immune axis function [23], [38], [39]. Chronic stress has also been shown to alter TLR expression in immune cells [20], [19]. This led us to investigate if this might result in changes in the expression of TLRs in the colonic mucosa. Comparing mRNA levels, we see a significant difference in the levels of TLR3, the receptor for dsRNA [40], in the proximal (p = 0.0006) and the distal colonic mucosa (p = 0.009) (Figure 1c & 1d), TLR4 (Figure 1e & 1f), the receptor for LPS, [41] in the proximal (p = 0.0035) and the distal colonic mucosa (p<0.0001) & TLR5 (Figure 1g & 1h), the receptor for flagellin, [42] in the proximal (p = 0.0007) and the distal colonic mucosa (p<0.0001). While TLR8 was significantly increased in the proximal but not the distal colon (p = 0.0359) (Figure 2c). No significant differences were noted for TLR2, 7, 9 & 10 while TLR6 could not be detected in all samples in both rat strains.

### TLR Expression in Wistar Kyoto (WKY) Compared with Sprague-Dawley (SD) Tissues

**Figure 3:**
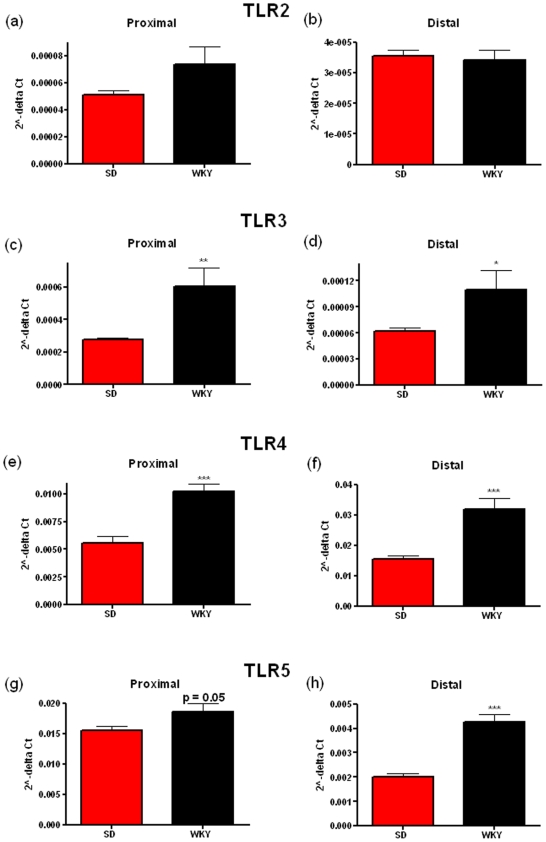
Relative levels of Toll-like Receptor (TLR) mRNA in the distal and proximal colonic mucosa of Wistar-Kyoto (WKY) and Sprague-Dawley (SD) rats. TLR2 (a, b), TLR3 (c, d), TLR4 (e, f) & TLR5 (g, h) mRNA levels are shown in both the proximal (a, c, e & g) and the distal (b, d, f & h) colon. Data was normalized to β-Actin and expressed using the 2^−ΔCt^ method. (** indicates p<0.01, *** indicates p<0.001).

**Figure 4:**
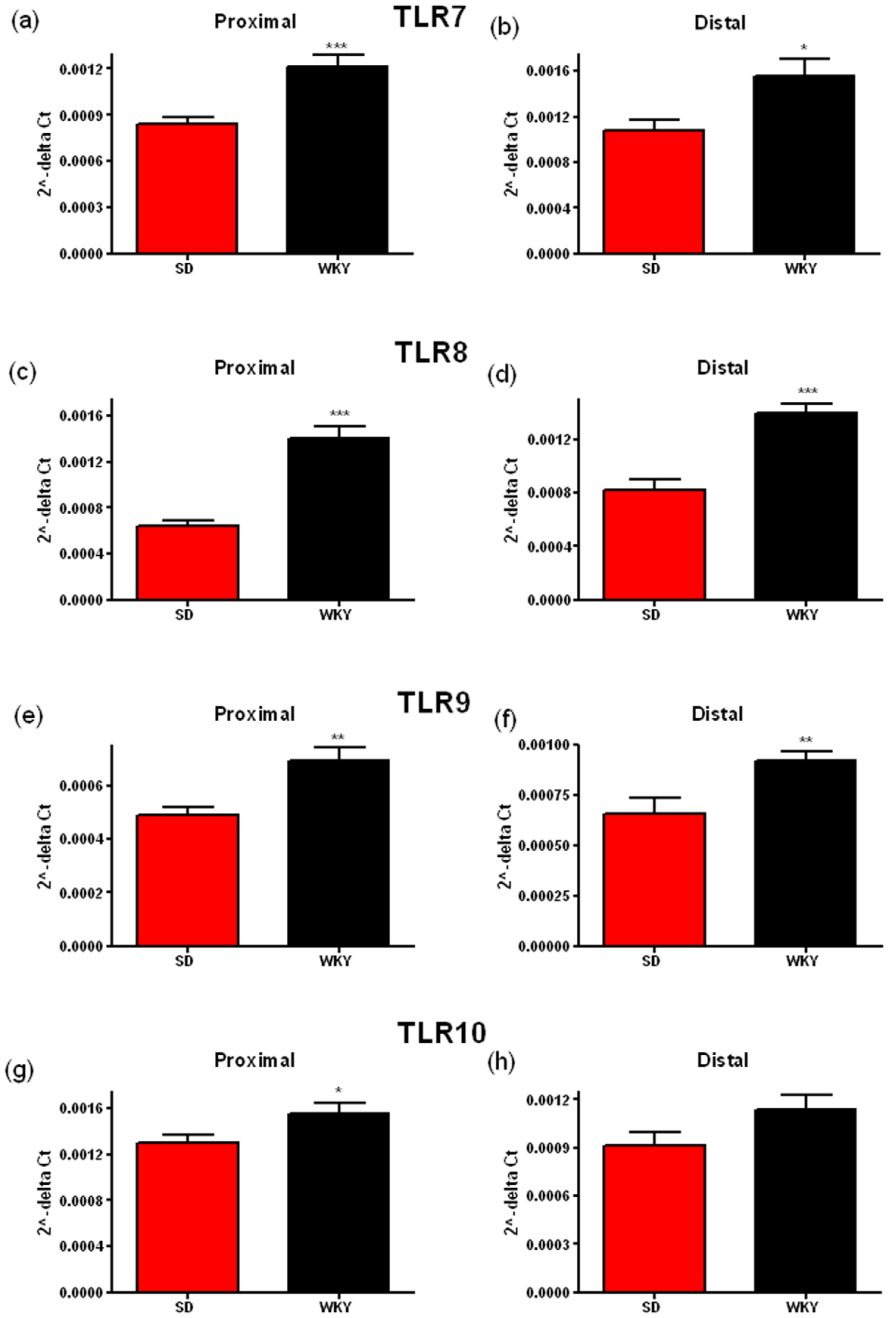
Relative levels of Toll-like Receptor (TLR) mRNA in the distal and proximal colonic mucosa of Wistar-Kyoto (WKY) and Sprague-Dawley (SD) rats. TLR7 (a, b), TLR8 (c, d), TLR9 (e, f) & TLR10 (g, h) mRNA levels are shown in both the proximal (a, c, e & g) and the distal (b, d, f & h) colon. Data was normalized to β-Actin and expressed using the 2^−ΔCt^ method. (** indicates p<0.01, *** indicates p<0.001).

The WKY rat also displays visceral hypersensitivity [30] and a highly anxious phenotype [39]. In comparison to normo-anxious SD rats. WKY animals have increased mRNA expression of TLR3 in the proximal (p = 0.0072) and the distal colonic mucosa (p = 0.00477) (Figure 3 c & 3d), TLR4 in the proximal (p<0.0001) and the distal colonic mucosa (p<0.0001) (Figure 3e & 3f) and TLR5 in the proximal (p = 0.05) and the distal colonic mucosa (p<0.0001) (Figure 3g & 3h). Moreover the expression of TLR7 in the proximal (p = 0.0004) and the distal colonic mucosa (p = 0.0203) (Figure 4a & 4b), TLR8 in the proximal (p<0.0001) and the distal colonic mucosa (p<0.0001) (Figure 4c & 4d), TLR9 in the proximal (p = 0.0015) and the distal colonic mucosa (p = 0.007) (Figure 4e & 4f) and TLR10 in the proximal colonic mucosa only (p = 0.0331) (Figure 4g) was greater when compared to the SD colonic mucosa. No significant differences in expression were found for TLR2 (Figure 3a & 3b) while as before, TLR6 could not be detected in all samples in both strains. The WKY rat strain appears to show differences compared to the SD strain in the same TLRs as the MS versus NS rats but also in several other TLRs, namely TLR7, 8, 9, 10. The functional significance of this is not known at present. TLR7 is known to bind to ssRNA from viruses [43] and TLR9 is known to bind to methylated DNA from bacteria and viruses [44] while there is no known ligand for TLR10 at present. Increases in these TLRs may result in increased inflammatory cytokine production and subsequent release into the circulation [45].

## Discussion

IBS is characterized by recurrent abdominal pain or discomfort at least 3 days per month for the past three months, associated with improvement of symptoms with defecation, onset associated with a change in the frequency and form/appearance of stool [46]. The syndrome has a worldwide prevalence of 10 to 15% [1], and chronic stress and anxiety have been thought to be major contributors to some of the symptoms of IBS [47]. There is an ongoing need to develop more models of irritable bowel syndrome in mice and rats to investigate the effects of chronic stress on the physiology of the gut because of the current lack of pharmacological treatments and complete understanding of the underlying causes. Two proposed rat models of IBS, the MS and WKY models used in this study have been shown to display changes in brain, gut and immune function that are relevant to the manifestation of IBS symptomatology [23], [30], [39].

At present there is a lack of reliable biomarkers in IBS patients [48]. It has been suggested that plasma cytokines maybe a putative biomarker for IBS. Pro-inflammatory cytokines such as IL-6, the IL-6 soluble receptor and IL-8 were all found to be elevated in IBS patients [7]. The increase in IL-6 and IL-8 may be linked to muscarinic receptor activity [49]. Another study has also found similar results, where IBS patients were shown to have increased levels of TNFα, IL-1β, IL6 at baseline and higher levels of LPS-induced IL-6 released in proximity or into circulation [10]. Previous work has shown that there are increased numbers of CD3+ and CD25+ in the lamina propria of IBS patients [50]. In addition, recent studies have also reported B-cell [51] and T-cell [52] activation in IBS patients suggesting aberrant immune activation. However, the origin of the cytokine release is not fully understood. One possible source of pro-inflammatory cytokine release is via activation of TLRs [45].

In this study, we have focussed on the expression of TLRs in the colonic mucosa, TLRs are key inducers of inflammation and to our knowledge, and this study shows for the first time that chronic stress in two animal models may affect the regulation of TLR expression in the colonic mucosa. In particular we demonstrate that both models display changes in the mRNA expression of TLR3, 4 and 5. As the ligands for these TLRs are both viral and bacterial this indicates that the stress models used in these experiments does not cause upregulation of any particular sub group of TLRs but results in a general increase in the level of these immune receptors. The upregulation of TLRs 4 and 5 suggests a potential increase in sensitivity to the presence of bacteria in the gut resulting in increases in cytokine production. Further studies will be needed to clarify if this is indeed the case. Interestingly, the increases seen in the WKY rat are somewhat different to the MS rats when both are compared to respective controls, indicating that these qualitatively different stressful states may differentially affect the expression of members of the innate immune receptors. Detailed comparisons between these two models may allow us to investigate further what factors may cause this differential expression. Possible reasons for these differences may lie in differences in neuroendocrine responses of the strains or the resident microbiota. We have shown that the MS model has different populations of microbiota in the gut compared to NS rats [23]. Studies analysing the microbiota in the WKY rat are ongoing. It is not known at present whether it is stress-induced changes in innate immune receptors facilitates the propagation of certain populations of bacteria or stress-induced changes in microbiota that lead to changes in innate immune receptor expression. Aside from the GI tract previous studies have shown that chronic stress may affect the expression of certain TLRs on circulating immune cells [19], [20] and our current data extend such observations to the GI colonic mucosae. It is not certain if other stress models share a similar pattern of expression as the ones mentioned in this study. We are unaware of other studies examining the immune effects of other chronic stress models in the GI tract but this is currently under investigation by our group.

It is not known at present what mechanism causes the increased expression of certain TLRs in the colonic mucosae of the MS and WKY rats. Stress, especially chronic stress is known to increase levels of glucocorticoids in addition to catecholamines, growth hormone and prolactin, all of which, have different effects on the immune system [53]. Corticosterone is elevated basally in the MS model [23] and shows a sustained increase following stress in the WKY model [54]. Glucocorticoids appear to be able to regulate the expression of certain TLRs. Of note it has been shown that glucocorticoids may synergize with either IL-1β [55], TNFα [56] or *H. influenzae* [57] to enhance the expression of TLR2 in human colonic or alveolar epithelial cells. A similar mechanism may exist in the stress models examined here with both showing greater stress-induced levels of glucocorticoids. Other mediators have been mentioned to affect TLR expression including catecholamines such as noradrenaline [58]. Infection with the obligate parasite *T. gondii* has been shown to increase expression of certain TLRs (TLR2, 4, 9 & 11 (murine)) while cold water stress following infection seems to decrease the expression of the same TLRs [58]. Noradrenaline treatment increased basal levels of certain TLRs while decreasing their expression in epithelial cells following *T. gondii* infection [58]. This may have relevance to IBS as catecholaminergic activity is often altered in such patients [59]. Moreover, in addition to bacterial components, fatty acids [60], [61] and opioids [62] have recently been shown to interact with TLR4 at least. These are also possibly other as yet unknown ligands that may interact with TLRs and result in the increase in cytokines seen in IBS patients

Another key question arising from this study is whether the changes in TLR expression are a state or trait marker of IBS-related symptomatology. Both these animal display visceral hypersensitivity in response to colorectal distension [23], [30]. TLRs may play an important role in visceral pain by indirectly increasing pro-inflammatory cytokine expression. It has been shown in models of neuropathic pain, where there is spinal nerve axotomy, genetic deletion of TLR2 [63], TLR3 [64] and TLR4 [65] results in reduced hypersensitivity and allodynia. More recently a TLR4 antagonist showed efficacy in a mouse peripheral nerve injury model [66]. The primary mechanism suggested in these studies is that pain results from spinal glial activation. Recently, it has been demonstrated that spinal microglia may play a key role in visceral hypersensitivity in a rat chronic stress model [67]. Inflammatory cytokine production from TLR activation may result in activation of glial cells on spinal nerves that innervate the gut.

In conclusion we have shown alterations in TLR expression levels in two models of stress which display symptoms relevant to IBS. Future studies will hopefully answer what specific chain of events causes the selective increase in TLR expression, how these are manifested at the protein level and what the functional significance of these increases are in terms of symptom manifestation in IBS. The future availability of high-quality specific antibodies targeting the various rodent TLRs would also be a major assistance in such exploits. Moreover, future investigations comparing the changes observed herein with those from other in vivo models of GI dysfunction (e.g. colitis) and/or stress are also warranted. Finally the translation of such findings into the clinical setting may pave the way for a novel biomarker of IBS where there is an immense medical need [48].
